# m^6^A Regulator-Based Exosomal Gene Methylation Modification Patterns Identify Distinct Microenvironment Characterization and Predict Immunotherapeutic Responses in Colon Cancer

**DOI:** 10.1155/2022/9451480

**Published:** 2022-08-22

**Authors:** Junjie Zheng, Panpan Feng, Dawei Chen, Cuiyu Zhang, Yuge Ji, Yanting Liu, Xiaohua Fan, Jingxin Li

**Affiliations:** ^1^Department of Physiology, School of Basic Medical Sciences, Cheeloo College of Medicine, Shandong University, Jinan, Shandong 250012, China; ^2^Department of Hematology, Qilu Hospital of Shandong University, Jinan, Shandong 250012, China; ^3^Laboratory of Medical Chemistry, Interdisciplinary Cluster for Applied Genoproteomics, GIGA-Stem Cells, Faculty of Medicine, University of Liège, CHU, Sart-Tilman, Liège 4000, Belgium; ^4^State Key Laboratory of Cellular Stress Biology, School of Life Sciences, Xiamen University, Xiamen, Fujian 361101, China; ^5^Department of Rehabilitation Medicine, Shandong Provincial Hospital Affiliated to Shandong First Medical University, Jinan, Shandong 250021, China

## Abstract

Recent studies have highlighted the biological significance of exosomes and m^6^A modifications in immunity. Nonetheless, it remains unclear whether the m^6^A modification gene in exosomes of body fluid has potential roles in the tumor microenvironment (TME). Herein, we identified three different m^6^A-related exosomal gene modification patterns based on 59 m^6^A-related exosomal genes, which instructed distinguishing characteristics of TME in colon cancer (CC). We demonstrated that these patterns could predict the stage of tumor inflammation, subtypes, genetic variation, and patient prognosis. Furthermore, we developed a scoring mode—m^6^A-related exosomal gene score (MREGS)—by detecting the level of m^6^A modification in exosomes to classify immune phenotypes. Low MREGS, characterized by prominent survival and immune activation, was linked to a better response to anti-PDL1 immunotherapy. In contrast, the higher MREGS group displayed remarkable stromal activation, high activity of innate immunocytes, and a lower survival rate. Hence, this work provides a novel approach for evaluating TME cell infiltration in colon cancer and guiding more effective immunotherapy strategies.

## 1. Introduction

More than 160 RNA chemical modifications have been identified, which greatly influence RNA function [1]. Several types of RNA modifications of eukaryotic mRNAs containing m^1^A, m^6^A, and m^5^C have been reported. Moreover, it is well known that m^6^A is the most commonly found in posttranscriptional modification of mRNA in most eukaryotes [2]. m^6^A methylation is reversible, which is similar to DNA and histone methylation in eukaryote cells, which were regulated by methyltransferases (writers), demethylases (erasers), and binding proteins (readers) [3]. The formation of m^6^A methylation is established by “writers” such as RBM15, ZC3H13, METTL3, METTL14, and KIAA1429, whereas removing m^6^A methylation is regulated by “erasers” FTO and ALKBH5. In addition, a group of specific RNA-binding proteins composed of YTHDF1/2/3, YTHDC1/2, HNRNPA2B1, LRPPRC, FMR1, CBLL1, and ELAVL1 can recognize the m^6^A motif, thus affecting m^6^A functions [4]. Recent studies revealed the interactions between TME immune cell infiltration and tumor m^6^A modification. These studies showed that tumor m^6^A methylation modification played in TME immune cell-infiltration characterization in various solid tumors, such as gastric cancer, head and neck cancer, and papillary thyroid carcinoma [5–7]. However, detailed roles and mechanisms of m^6^A methylation modification in TME of CC remain unclear.

Exosome undoubtedly was one of the most pathbreaking scientific achievements in cellular biology. Exosomes were first discovered in reticulocytes relevant to transferrin in 1983, and their size ranged from 50 to 110 nm, influencing cell management and intercellular crosstalk [8]. Many studies showed that tremendous alterations existed in exosomes between normal and cancer cells. The exosome release rate and contents (microRNAs) are pronouncedly increased in cancer cells. The same results were observed in cancer cell lines [9]. The tumor microenvironment (TME) is the cellular environment in which the tumor develops, including various cell types, extracellular matrix (ECM), growth factors, proteolytic enzymes and their inhibitors, and signaling molecules [10]. As emerging components of the tumor-host interaction, tumor-derived exosomes are involved in TME formation. The contents of exosomes are hinged on their donor cells, and there are various ways in which exosomes transfer messages to target sites in the microenvironment, such as proteins, RNA, and DNA. Tumor-derived exosomes carried and transferred a variety of biomolecules that could mediate intercellular communication, promote tumorigenesis and metastasis, and regulate the microenvironment and immune system [11, 12]. However, little is known about the role of epigenetic modification, especially for m^6^A methylation, of exosomal contents in TME.

Immunotherapy is a hotly debated topic in oncology. In recent years, the arrival of several innovative monoclonal antibodies used for immunotherapy has revolutionized cancer treatment for a wide range of solid tumors. Unfortunately, CC is one of the most common malignancies and remains the primary cause of cancer death worldwide [13, 14]. Despite many advances in systemic therapies, approximately 86% of patients with advanced stages die within five years of diagnosis. Recently, current immunotherapies represented by specific immune checkpoint inhibitors (ICIs), such as anti-CTLA-4 and anti-PD-1/L1, have achieved a marked durable response in CC treatment [15, 16]. Evaluating immune infiltration based on the characteristics of TME constitutes a critical approach to predicting the response to existing ICIs and developing novel immunotherapeutic strategies [17, 18].

This study systematically evaluated the m^6^A regulator-based exosomal RNA modification pattern in CC and identified the association between the three patterns and TME cell-infiltrating characteristics. Moreover, we demonstrated that the three patterns could predict stages of tumor inflammation, subtypes, genetic variation, and patient prognosis. In addition, we developed a scoring mode—m^6^A-related exosomal gene score (MREGS)—by detecting the level of m^6^A modification in exosomes to classify immune phenotypes, which could predict the prognosis, immunity state, and immunotherapy response to anti-PDL1. Therefore, we developed a new scoring system to quantify the m^6^A-based exosomal RNA modification patterns in individual CC patients.

## 2. Materials and Methods

### 2.1. Colon Cancer Dataset Source and Preprocessing

Public gene-expression data and complete clinical annotation were searched in the Gene Expression Omnibus (GEO) and the Cancer Genome Atlas (TCGA) database. Patients without survival information were removed from further evaluation. Seven eligible CC cohorts (GSE17536, GSE29621, GSE33113, GSE37892, GSE38832, GSE39582, and TCGA-COAD) were gathered in this study for further analysis. For microarray data from Affymetrix®, we downloaded the raw “CEL” files and adopted a robust multiarray averaging method with the affy and simplified packages to perform background adjustment and quantile normalization. The normalized matrix files were directly downloaded for microarray data from other platforms. For datasets in TCGA, RNA sequencing data (FPKM value) of gene expression were downloaded from the Genomic Data Commons (GDC, https://portal.gdc.cancer.gov/) using R package TCGAbiolinks, which was developed explicitly for integrative analysis with GDC data. Then, FPKM values were transformed into transcripts per kilobase million (TPM) values. Batch effects from nonbiological technical biases were corrected using “ComBat” algorithm of the sva package. The somatic mutation data were acquired from TCGA database. GSE39582 dataset from GEO was downloaded for copy number variation (CNV) analysis. The data were analyzed using R (version 3.6.1) and R Bioconductor packages.

### 2.2. Unsupervised Clustering for 59 m^6^A-Related Exosome Genes

A total of 59 m6A-related exosome genes were screened through the exoRBase database (http://www.exoRBase.org) and TCGA database (https://portal.gdc.cancer.gov/). Firstly, we analyzed the differentially expressed genes between CC and normal samples in the two databases and overlapped these two differential gene sets to obtain the intersecting genes (*P* < 0.05). Then, we performed the correlation analysis between these intersecting genes with 24 m^6^A regulators and obtained 59 m6A-related exosome genes (standard: *P* < 0.001, correlation coefficient ≥ 0.5). Unsupervised clustering analysis was applied to identify distinct m^6^A-related exosome gene modification patterns based on the expression of 59 m^6^A-related exosome genes and classify patients for further analysis. The consensus clustering algorithm determined the number of clusters and their stability. We used the ConsensusClusterPlus package to perform the above steps. Moreover, 1000 repetitions were conducted to guarantee the classification's stability [19].

### 2.3. Statistical Analysis

Correlations coefficients between TME-infiltrating immune cells and expression of m^6^A-related exosome genes were computed by Spearman and distance correlation analyses. One-way ANOVA and Kruskal-Wallis tests were used to conduct difference comparisons of three or more groups. Survminer R package was used to determine the cutoff point for each dataset subgroup based on the correlation between MREGS and patient survival. The “surv-cutpoint” function, which repeatedly tested all potential cutpoints for finding the maximum rank statistic, was applied to dichotomize MREGS, and then, patients were divided into high and low MREGS groups based on the maximally selected log-rank statistics to decrease the batch effect of calculation. The survival curves for the prognostic analysis were generated via Kaplan-Meier method, and log-rank tests were utilized to identify the significance of differences. We adopted a univariate Cox regression model to calculate the hazard ratios (HR) for m^6^A-related exosome genes and m^6^A phenotype-related exosome genes. The independent prognostic factors were ascertained through a multivariable Cox regression model. Patients with detailed clinical data were eligible for final multivariate prognostic analysis. The Forestplot R package was employed to visualize the results of multivariate prognostic analysis for MREGS in TCGA-COAD cohort. The specificity and sensitivity of MREGS were assessed through the receiver operating characteristic (ROC) curve, and the area under the curve (AUC) was quantified using pROC R package. The waterfall function of Maftools package was used to present the mutation landscape in patients with high and low MREGS subtypes in TCGA-COAD cohort. R package of RCircos was adopted to plot the copy number variation landscape of 59 m^6^A-related exosome genes in 23 pairs of chromosomes. All statistical *P* values were two sides, with *P* < 0.05 as statistically significant. All data processing was done in R 3.6.1 software.

Other detailed information for the materials and methods is provided in the supplementary materials.

## 3. Results

### 3.1. Landscape of Genetic Variation of m^6^A-Related Exosome Genes in Colon Cancer

**Figure 1:**
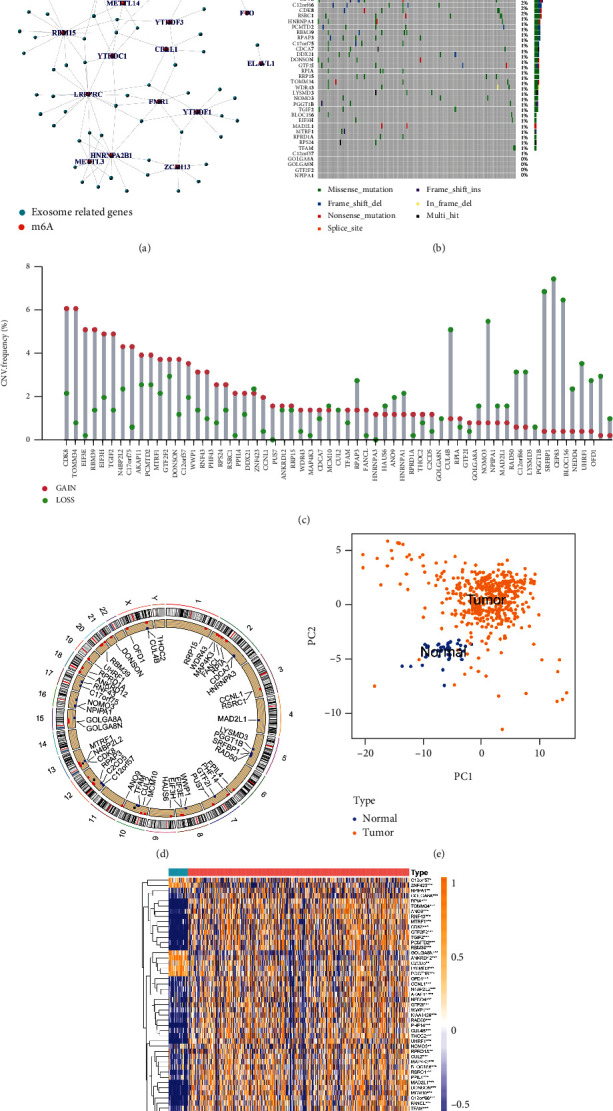
Landscape of genetic variation of m^6^A-related exosome gene in colon cancer. (a) m^6^A-related exosome gene. Exosome-related genes mean differential expression genes of serum exosome genes between patients with colon cancer and normal people. (b) The mutation frequency of 59 m^6^A-related exosome gene in 399 patients with colon cancer from TCGA-COAD cohort. Each column represented individual patients. The upper bar plots showed TMB; the number on the right indicated the mutation frequency in each regulator. The right bar plots showed the proportion of each variant type. The stacked bar plots below showed fraction of conversions in each sample. (c) The CNV variation frequency of m^6^A-related exosome gene in the GSE39582 cohort. The height of the column represented the alteration frequency. The deletion frequency, green dot. The amplification frequency, red dot. (d) The location of CNV alteration of m^6^A-related exosome gene on 23 chromosomes using the GSE39582 cohort. (e) Principal component analysis for the expression profiles of 59 m^6^A-related exosome gene to distinguish tumors from normal samples in the GSE39582 cohort. Two subgroups without intersection were identified, indicating the tumors and normal samples were well distinguished based on the expression profiles of m^6^A-related exosome gene. Tumors were marked with yellow, and normal samples marked with blue.

We selected the differential expression genes of exosomes from CC patients' serum and normal serum, named exosome-related genes. Next, those genes associated with m^6^A regulators were sorted out, and we obtained 59 m^6^A-related exosome genes. The relationship between m^6^A regulators and exosome-related genes is shown in Figure 1(a). It is commonly acknowledged that somatic mutations and CNV (copy number variation) are linked with cancers [20, 21]. Therefore, we prefer to acquire a preliminary knowledge of the frequency of somatic variation and CNV of 59 m^6^A-related exosome genes in CC patients. In 399 patients, mutations of m^6^A-related exosome genes occurred in 159 patients, accounting for 39.85%. The histogram displayed that RNF43 was ranked first on mutation frequency and ZNF423 subsequently followed. However, no mutations exist in TFAM, ALKBH5, C12orf57, GOLGA8A, GOLGA8N, GTF2F2, and NPIPA1 (Figure 1(b)). The following analyses showed the prominent mutation cooccurrence relationship including ANKRD12 and RNF43, RBM39, and CUL2, besides THOC2 and C2CD5 (Figure S1a). By observing distinct CNV alteration of 59 m^6^A-related exosome genes, it is obvious that copy number amplification occupied a majority of alterations, whereas CNV deletion happened in LYSMD3, PGGT1B, and SRFBP1 (Figure 1(c)). The analysis of somatic mutation and CNV suggested that the chosen 59 genes, which were delivered by exosomes, probably derived from tumors. Subsequently, Figure 1(d) exposes the mutated site of the m^6^A-related exosome genes on CC patients' chromosomes. Then, we could tell CC patients and normal samples apart based on different expressions of 59 m^6^A-related exosome genes (Figure 1(e)). To assure whether the expression of m^6^A-related exosome genes was affected by the aforementioned genetic variations in CC patients, we found that the changes of CNV could exert prodigious influence on perturbations of m^6^A-related exosome gene expression through studying mRNA expression of these 59 genes between normal and CC samples. In addition, the m^6^A-related exosome genes with amplified CNV in CC tissues showed a remarkably higher expression versus normal colon tissues such as TOMM34 and RBM39 (Figures 1(c) and 1(f)). No matter in the transcriptome or genomics, there was a significant difference between patients and normal people in 59 m^6^A-related exosome genes. The above results suggested that the expression imbalance of the m^6^A-related exosome genes positively influence the CC genesis and development.

### 3.2. m^6^A Methylation Modification Patterns Mediated by 59 m^6^A-Related Exosome Genes

**Figure 2:**
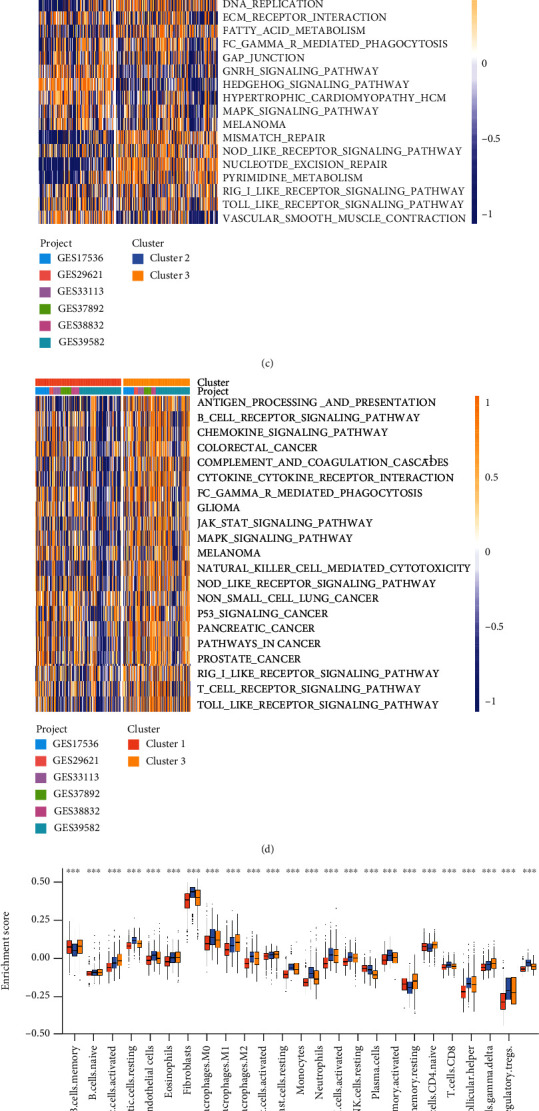
Biological characteristics and TME cell infiltration of m^6^A-related exosome gene modification patterns. (a) The interaction between m^6^A-related exosome genes in colon cancer. (b) Survival analyses for the three m^6^A-related exosome gene modification patterns based on 1066 patients with colon cancer from six GEO cohorts (GSE17536, GSE29621, GSE33113, GSE37892, GSE38832, and GSE39582) including 442 cases in cluster 1, 263 cases in cluster 2, and 361 cases in cluster 3. Kaplan-Meier curves with log-rank *P* value 0.001 showed a significant survival difference among three m^6^A-related exosome gene modification patterns. (c, d) GSVA enrichment analysis showing the activation states of biological pathways in distinct m^6^A-related exosome gene modification patterns. The heat map was used to visualize these biological processes, and yellow represented activated pathways, and blue represented inhibited pathways. The colon cancer cohorts were used as sample annotations. (c) Cluster 2 vs. cluster 3; (d) cluster 1 vs. cluster 3. (e) The abundance of each TME-infiltrating cell in three m^6^A-related exosome gene modification patterns. The upper and lower ends of the boxes represented interquartile range of values. The lines in the boxes represented median value, and black dots showed outliers. (f) Differences in CD8^+^ T effector and stroma-activated pathways including EMT, TGF*β*, and angiogenesis pathways among three distinct m^6^A-related exosome gene modification patterns. The statistical differences among three modification patterns were tested by the one-way ANOVA test. The asterisks represented the statistical *P* value (^∗^*P* < 0.05; ^∗∗^*P* < 0.01; ^∗∗∗^*P* < 0.001).

We first enrolled six GEO datasets (GSE17536, GSE29621, GSE33113, GSE37892, GSE38832, and GSE39582) which contained clinical data and overall survival (OS) data into one metacohort. The comprehensive landscape of m^6^A-related exosome gene interactions for CC patients was depicted with the m^6^A-related exosome gene network (Figure 2(a)). The relationship between those exosome-related genes is shown in Figure S1a, and the HR value of the genes is shown in Figure S1b. Then, according to the expression of 59 m^6^A-related exosome genes, we could divide CC patients into three qualitative m^6^A-related exosome gene modification patterns using the R package of ConsensusClusterPlus. Moreover, an unsupervised clustering algorithm distinguished three patterns (442 cases in pattern 1, 263 cases in pattern 2, and 361 cases in pattern 3). Finally, these patterns were called clusters 1-3 (Figure S2). The tremendous survival preponderance in cluster 1 was disclosed by prognostic analysis for three modification subtypes (Figure 2(b)).

### 3.3. TME Cell Infiltration Characteristics in Distinct m^6^A-Related Exosome Gene Modification Patterns

Because the biological behavior between the three m^6^A modification patterns was not completely understood, we performed gene set variation analysis (GSVA) of hallmark gene sets in six GEO datasets (GSE17536, GSE29621, GSE33113, GSE37892, GSE38832, and GSE39582). Cluster 3 was markedly enriched in mismatch repair, pyrimidine metabolism, and Toll-like receptor signaling pathway (Figure 2(c)). However, we observed that innate immune cells such as NK cells, macrophages, and MDSC were conspicuously enriched in TME cell infiltration of cluster 2 (Figure 2(e)). Previous studies defined tumors as three phenotypes: immune-excluded, immune-inflamed, and immune-desert. Furthermore, the immune-excluded phenotype was characteristic of plentiful immune cells around the tumor cell nest, yet the tumor capsules showed powerful protectivity from immune cell penetration. Moreover, it demonstrated that poor efficiency in tumor penetration of immune cells might be caused by stromal activation [22]. Hence, we hypothesized that the antitumor function of immune cells in cluster 2 was restricted by stromal activation. Subsequent analyses confirm that the activation of epithelial-mesenchymal transition (EMT), transforming growth factor-beta (TGF*β*), and angiogenesis pathways, which are relevant to stromal activation in the tumor, were remarkably increased in cluster 2 (Figure 2(f)). Because of the mentioned analysis, cluster 2 which featured innate immune cell infiltration and stromal activation corresponded with the immune-excluded subtype. Cluster 3 which was featured with specific immune cell infiltration and immune activation corresponded with the immune-inflamed subtype. The comparison between clusters 1 and 3 revealed that cluster 1 was not concentrating on antigen processing and presentation, chemokine signaling pathway, and cytokine-cytokine receptor interaction associated with adaptive immune. Instead, cluster 1 had immunological ignorance, corresponding to the immune-desert phenotype (Figures 2(d)–2(f)). The mutual effect between each tumor-infiltrating immune cell type and each m^6^A-related exosome gene was then demonstrated using Spearman's correlation analysis (Figure S3).

### 3.4. m^6^A Methylation Modification Patterns in the GSE39582 Cohort

**Figure 3:**
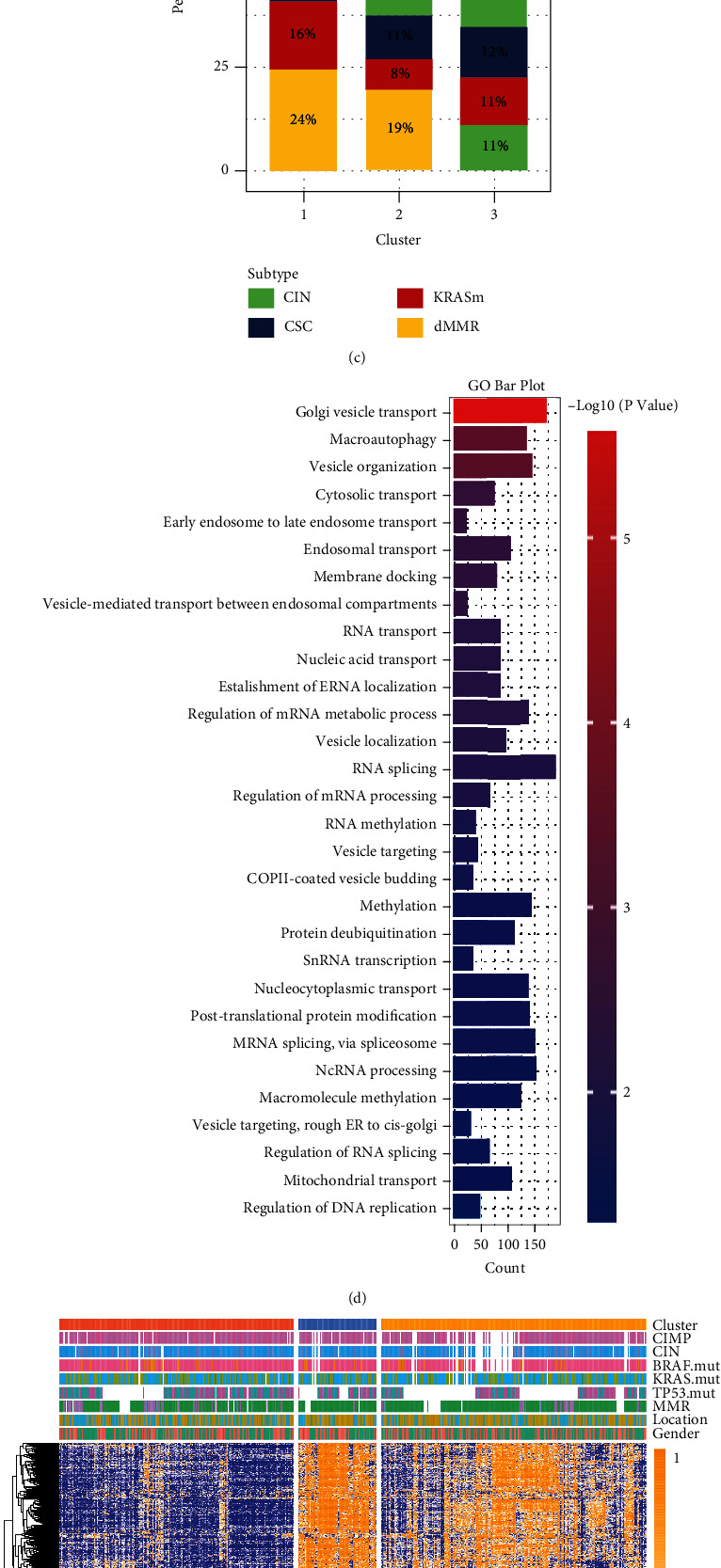
Transcriptome traits in m^6^A-related exosome gene modification patterns and unsupervised clustering based on 3787 signatures. (a) Unsupervised clustering of 59 m^6^A-related exosome genes in the GSE39582 colon cancer cohort. The cluster, CC molecular subtypes, gene mutation, location, and gender were used as patient annotations. Yellow represented high expression of regulators, and blue represented low expression. (b) Principal component analysis for the transcriptome profiles of three m^6^A-related exosome gene modification patterns, showing a remarkable difference on transcriptome between different modification patterns. (c) The proportion of GSE39582 molecular subtypes in the three modification patterns. CIN subtype, green; CSC subtype, blue; KRASm subtype, red; dMMR subtype, yellow. (d) Functional annotation for m^6^A-related exosome gene using GO enrichment analysis. The color depth of the bar plots represented the number of genes enriched. (e) Unsupervised clustering of overlapping m^6^A-related exosome gene phenotype in GSE39582 cohorts to classify patients into different genomic subtypes, termed as m^6^A-related exosome gene clusters A-C, respectively. The cluster, CC molecular subtypes, gene mutation, location, and gender were used as patient annotations. Yellow represented high expression of regulators, and blue represented low expression. (f) Kaplan-Meier curves indicated m^6^A-related exosome gene modification genomic phenotypes were markedly related to overall survival of 557 patients in the GSE39582 cohort, of which 213 cases were in gene cluster A, 85 cases in gene cluster B, and 259 cases in gene cluster C (*P* < 0.0001, log-rank test). (g) The expression of 24 m^6^A regulators in three gene clusters. The upper and lower ends of the boxes represented interquartile range of values. The lines in the boxes represented median value, and black dots showed outliers. The asterisks represented the statistical *P* value (^∗^*P* < 0.05; ^∗∗^*P* < 0.01; ^∗∗∗^*P* < 0.001). The one-way ANOVA test was used to test the statistical differences among three gene clusters.

Next, we focused on the GSE39582 cohort to comprehensively understand m^6^A-related exosome gene modification patterns in numerous clinical cases. Consequently, using an unsupervised clustering algorithm, three patterns were distinctly classified in the GSE39582 cohort (Figures 3(a) and S4a-d). Also, among three different m^6^A-related exosome gene modification patterns, the major difference was shown on the principal component analysis (PCA) scatter diagram. (Figure 3(b)). Marisa et al. innovatively classified patients who were suffering from CC into four dominant molecular subtypes including CSC (cancer stem cell), CIN (chromosome instability), KRASm (KRAS mutant), and dMMR (defective mismatch repair). Moreover, they concluded that CIN is associated with upregulation of the EMT pathways while dMMR is associated with upregulation of the immune pathways and cell proliferation. However, EMT is downregulated in KRASm subtype [23]. Consistent with the previous findings, CIN subtype patients were divided into clusters 2 and 3, while dMMR subtype predominantly focused on cluster 1 (Figure 3(c)). Furthermore, prognostic analysis depicted that cluster 1 had a significant survival rate advantage compared to clusters 2 and 3 (Figure S4e). Also, we found differential expression of m^6^A regulators among three m^6^A methylation modification patterns (Figure S4f).

### 3.5. Generation of m^6^A-Related Exosome Gene Signatures and Functional Annotation

To discover the unknown biological functioning of m^6^A-related exosome gene modification patterns, we used the limma package to identify 3787 m^6^A-related exosome gene phenotype-related differential expression genes (DEGs) (Figure S4g). In addition, GO enrichment analysis for differential expression genes was exhibited by the clusterProfiler package. Consequently, 3787 selected DEGs, regarded as m^6^A-related exosome gene signatures, were denoted as the pivotally salient indicator of three m^6^A-related exosome gene modification patterns. These signatures performed a significant abundance of biological processes that corresponded with the formation of the exosome, m^6^A modification, and RNA transport, which verified that tumors could deliver m^6^A-methylated RNA by exosomes to target cells (Figure 3(d)). Owing to the absence of validation for this supervision mechanism, we applied unsupervised clustering analysis based on the selected 3787 signatures to divide patients into different genomic subtypes. As a result, we obtained three newly established distinct subtypes called m^6^A-related exosome gene clusters A-C (Figures S5a-d and 3(e)). This phenomenon ensured again that there were three distinct m^6^A-related exosome gene modification patterns in CC. Patients with colon cancer in gene cluster A (213 patients) ranked first in the prognosis analysis. In contrast, the worst survival rate was shown in gene cluster B, with 85 patients classified. In total, 259 patients were classified in gene cluster C, regarded as an intermediate prognosis (Figure 3(f)). Furthermore, among three gene clusters (A-C), it was discovered that the prodigious difference in 24 m^6^A regulator expressions was consistent with the anticipated outcome of m^6^A methylation modification patterns (Figure 3(g)).

### 3.6. Characteristics of Clinical and Transcriptome Traits in m^6^A-Related Exosome Gene Phenotypes

To figure out the function of m^6^A-related exosome gene phenotypes in the TME immunity moderation, we chose some cytokines and chemokines from three groups, which were gathered from published articles online. First, TGRB1, SMAD9, TWIST1, CLDN3, TGFBR2, ACTA2, COL4A1, ZEB1, and VIM are denoted as the transcripts of the transforming growth factor (TGF*β*)/EMT pathway. Second, PD-L1, CTLA-4, IDO1, LAG3, HAVCR2, PD-1, PD-L2, CD80, CD86, TIGIT, and TNFRSF9 were relevant to the transcripts of immune checkpoints. Furthermore, third, TNF, IFNG, TBX2, GZMB, CD8A, PRF1, GZMA, CXCL9, and CXCL10 were to be correlated with the pathways of immune activation [24, 25]. We considered gene cluster C was a stromal-activated group because of its increased mRNA expression of the TGF*β*/EMT pathway. However, transcriptional mRNAs correlating with immunity system activity were upregulated in gene cluster A, suggesting it probably was categorized as the immune-inflamed group (Figures S5f-5h). The next step was to pick out a portion of known signatures in CC patients to describe the function of m^6^A-related exosome signatures (Figure S5e). As expected, these box plots showed that gene cluster C featured higher stroma activity and cancer progression and metastasis, such as increased EMT and WNT target, while cell cycle, DNA replication, and mismatch repair were remarkably enhanced in gene cluster A.

**Figure 4:**
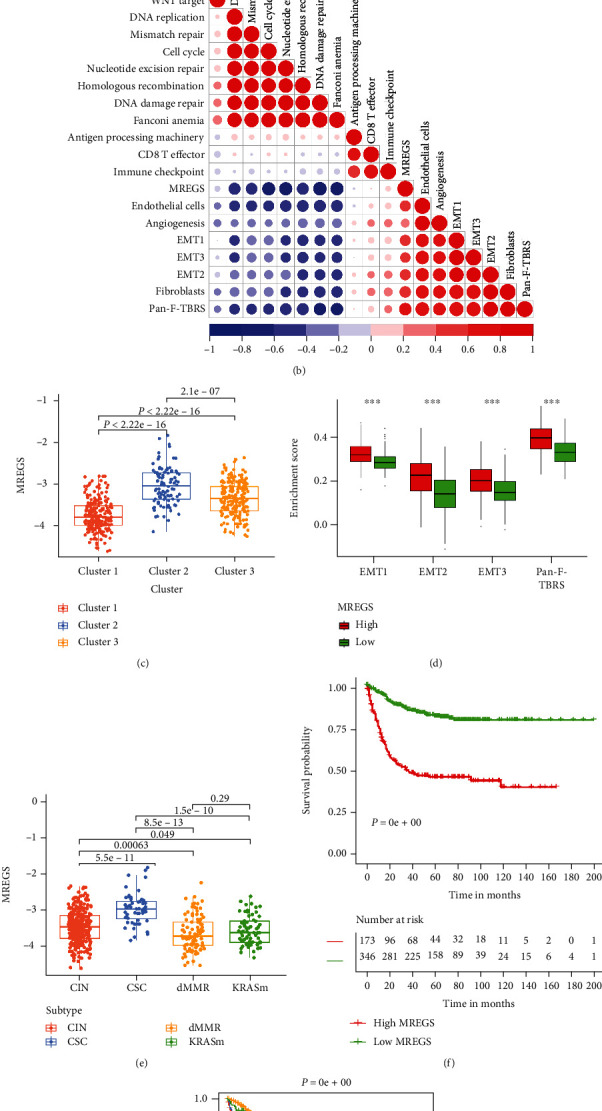
Characteristics of clinical and transcriptome traits in m^6^A-related exosome gene phenotypes. (a) Alluvial diagram showing the changes of clusters (1, 2, and 3), m^6^A-related exosome gene clusters (A, B, and C), GSE39582 molecular subtypes, and MREGS. (b) Correlations between MREGS and the known gene signatures in the GSE39582 cohort using the Spearman analysis. Negative correlation was marked with blue, and positive correlation with red. (c) Differences in MREGS among three gene clusters in the GSE39582 cohort. The Kruskal-Wallis test was used to compare the statistical difference between three m^6^A-related exosome gene clusters. (d) Differences in stroma-activated pathways between the high MREGS and low MREGS groups. EMT: epithelial-mesenchymal transition; Pan-F-TBRS: panfibroblast TGF*β* response signature. The upper and lower ends of the boxes represented interquartile range of values. The lines in the boxes represented median value. The asterisks represented the statistical *P* value (^∗^*P* < 0.05; ^∗∗^*P* < 0.01; ^∗∗∗^*P* < 0.001). (e) Differences in MREGS between different GSE39582 molecular subtypes. The Kruskal-Wallis test was used to compare the statistical difference between four GSE39582 molecular subtypes (*P* < 0.05). (f) Survival analyses for low (346 cases) and high (173 cases) MRGES groups in the GSE39582 cohort using Kaplan-Meier curves (*P* < 0.0001, log-rank test). (g) Survival analyses for subgroup patients stratified by both MREGS and treatment with adjuvant chemotherapy using Kaplan-Meier curves. H: high; L: low; ADJC; adjuvant chemotherapy (*P* < 0.05, log-rank test).

To more accurately predict the patterns of m^6^A methylation modification in a single tumor, we should use a different method to compensate for the error caused by the heterogeneity of each tumor. Hence, we developed a scoring scheme called the MREGS (m^6^A-related exosome gene score), based on the identified 3787 signature genes to calculate the score of individual CC patients. Because of the complicacy of m^6^A-related exosome gene modification, we used an alluvial diagram to observe the flow of data (Figure 4(a)). Furthermore, by exploring the relationship between MREGS and some recognized signatures, we could have more knowledge of m^6^A-related exosome gene signatures. Moreover, it was found that MREGS was positively associated with EMT- and TGF*β*-related pathway and angiogenesis, whereas it was negatively related to DNA damage repair and mismatch repair (Figure 4(b)). Also, there was a prodigious difference that existed in MREGS between m^6^A-related exosome gene clusters (clusters 1, 2, and 3) by the Kruskal-Wallis test. It displayed that cluster 2 corresponded with high MREGS but cluster 1 represented relatively low MREGS (Figure 4(c)). Additionally, as the activation of fibroblasts is dependent on TGF*β* secreted by immunocytes or cancer cells and IL-11 secreted by TGF*β*1-stimulated CAFs (cancer-associated fibroblasts) could enhance the survival rate and invasion ability of cancer cells [26, 27], we chose to analyze stromal activation-related and TGF*β* pathways. We found that fibroblasts' increased stromal activation and TGF*β* pathway activity corresponded with high MREGS (Figure 4(d)). Moreover, the dMMR subtype ranked last in MREGS, while the CSC subtype was the highest (Figure 4(e)). The mentioned description powerfully elucidated that low MREGS was associated with DNA damage repair and high MREGS was associated with stromal activation. Furthermore, we attempted to ascertain the significance of MREGS in forecasting patients' prognoses. Patients with low MREGS were superior to those with high MREGS survival rates (Figure 4(f)) because their 5-year survival rate was two times higher than those with high MREGS. Whether MREGS could act as a robust prognostic biomarker for colon cancer is the next question we were supposed to solve. Consequently, we used a multivariate Cox regression model analysis, which contained patients' gender, age, stages, tumor location, MMR status, and subtypes. We found that MREGS was interrelated with patients' age, stages, and MMR status which confirmed MREGS as a creditable and independent prognostic biomarker for evaluating patient outcomes (Figures S6a-b). Due to adjuvant chemotherapy (ADJC) being a standard treatment after colon cancer surgery, MREGS was designed to validate its predictive ability to the efficacy of ADJC in CC patients. We discovered that patients with low MREGS-ADJC displayed remarkable adverse reactions compared with patients who did not receive adjuvant chemotherapy. At the same time, ADJC positively impact patients with high MREGS. The high MREGS-ADJC patients displayed key therapeutic advantages compared with patients who did not receive adjuvant chemotherapy. The other obtained results indicated that low MREGS patients invariably displayed a tremendous survival advantage regardless of ADJC treatment (Figure 4(g)).

Moreover, according to mutation patterns, tumors can be classified into dMMR and pMMR. DMMR (defective mismatch repair) had more mutation burden than pMMR (proficient mismatch repair) [28]. In addition, we discussed the correlation between MREGS and molecular subtype and discovered that the pMMR subtype was distinctly related to higher MREGS. In addition, in the stage IV patients, there was a remarkable enhancement in MREGS compared to three other groups (Figure S6b). This is consistent with the result that the dMMR subtype was related to higher survival than the pMMR subtype. These phenomena illustrated that MREGS could also indirectly assess some clinical features such as MMR subtypes and clinical stage.

### 3.7. Characteristics of m^6^A-Related Exosome Gene Modification in TCGA Molecular Subtypes and Somatic Tumor Mutation

**Figure 5:**
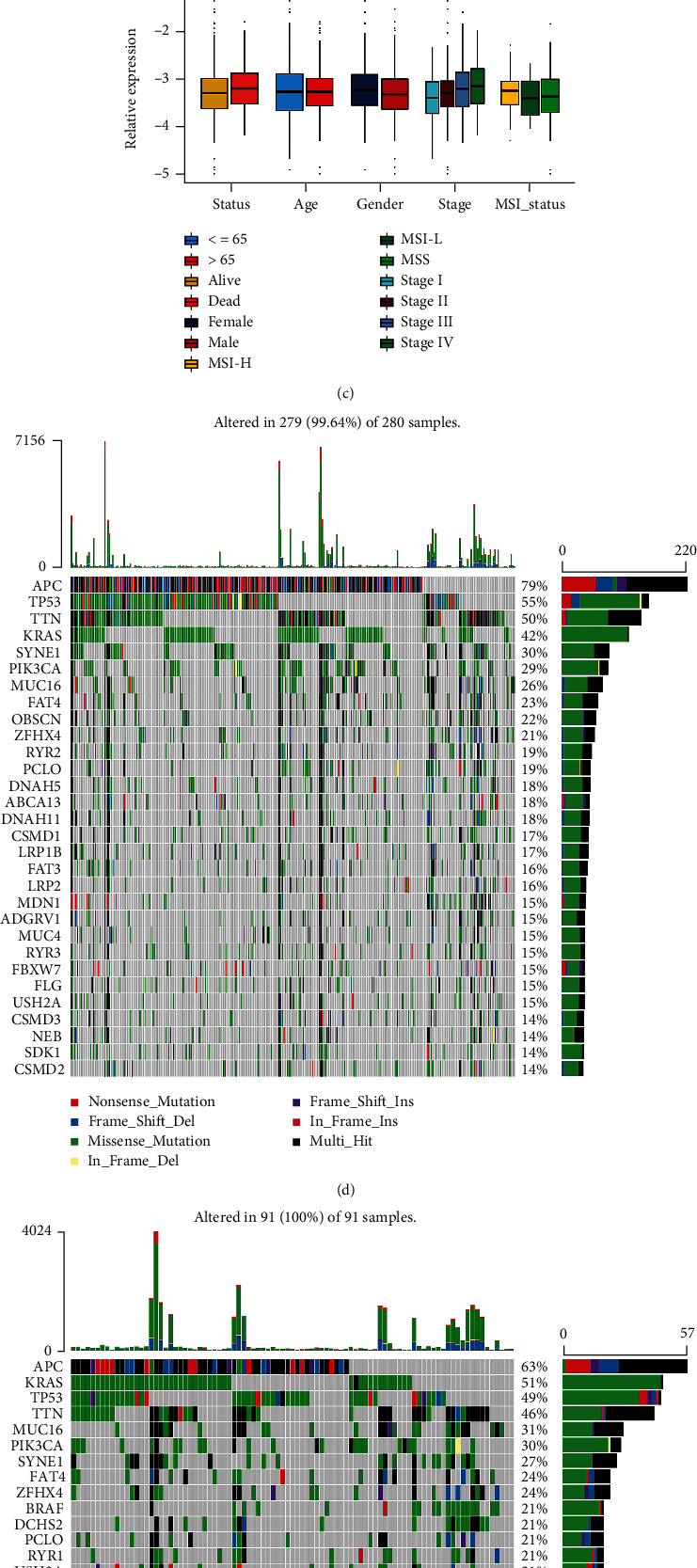
Characteristics of m^6^A-related exosome gene modification in TCGA molecular subtypes and tumor somatic mutation. (a) Survival analyses for low (331 cases) and high (99 cases) MREGS patient groups in TCGA-COAD cohort using Kaplan-Meier curves (*P* = 0.009, log-rank test). (b) Differences in MREGS between different TCGA-COAD molecular subtypes. The upper and lower ends of the boxes represented interquartile range of values. The lines in the boxes represented median value. The Kruskal-Wallis test was used to compare the statistical difference between four TCGA-COAD molecular subtypes. CIN: chromosomal instability; MSI: microsatellite instability; CIMP: CpG island methylator phenotype. (c) Differences in MREGS among different types. The upper and lower ends of the boxes represented interquartile range of values. The lines in the boxes represented median value. The asterisks represented the statistical *P* value (^∗^*P* < 0.05; ^∗∗^*P* < 0.01; ^∗∗∗^*P* < 0.001). MSS: microsatellite stable; MSI-H: high microsatellite instability; MSI-L: low microsatellite instability. (d, e) The waterfall plot of tumor somatic mutation established by those with high MREGS (d) and low MREGS (e). Each column represented individual patients. The upper bar plots showed TMB (tumor mutation burden); the number on the right indicated the mutation frequency in each gene. The right bar plots showed the proportion of each variant type.

To comprehensively study the characteristics of m^6^A-related exosome gene modification patterns, we introduced TCGA project. Three phenotypes classified by TCGA project are comprised chromosomal instability (CIN), invasive, and microsatellite instability (MSI). Next, the difference in MREGS among the three phenotypes was calculated. The higher MREGS was pronouncedly concentrated on CIN and had a shorter lifespan, whereas the invasive phenotype is relevant with lower MREGS, which was related to better survival (Figures 5(a) and 5(b)). Different stages indicated differential expression of m^6^A-related exosome gene in TCGA project (Figure 5(c)). Additionally, we used the Maftools software package to analyze the differences in the distribution of high MREGS and low MREGS somatic mutations in TCGA-COAD cohort. A more extensive tumor mutation burden was presented in the low MREGS patients than the high MREGS group (Figures 5(d) and 5(e)). Accumulated evidence demonstrated a potential connection between the enhanced survival rate of receiving PD-1/PD-L1 immunological therapy and higher somatic tumor mutation burden (TMB). Consequently, it was indirectly elucidated that clinical reactions to immunological treatments like anti-PD-1/PD-L1 drugs may depend on different m^6^A-related exosome gene modification patterns in tumors. Also, we confirmed MREGS as a reliable method to predict prognostic outcomes after immunotherapy. In both clinical trials and preclinical studies, higher TMB patients receiving immune checkpoint inhibitor treatment have a prominent superiority in survival rate and clinical response [29].

### 3.8. m^6^A-Related Exosome Gene Modification Patterns in the Role of Anti-PD-1/L1 Immunotherapy

**Figure 6:**
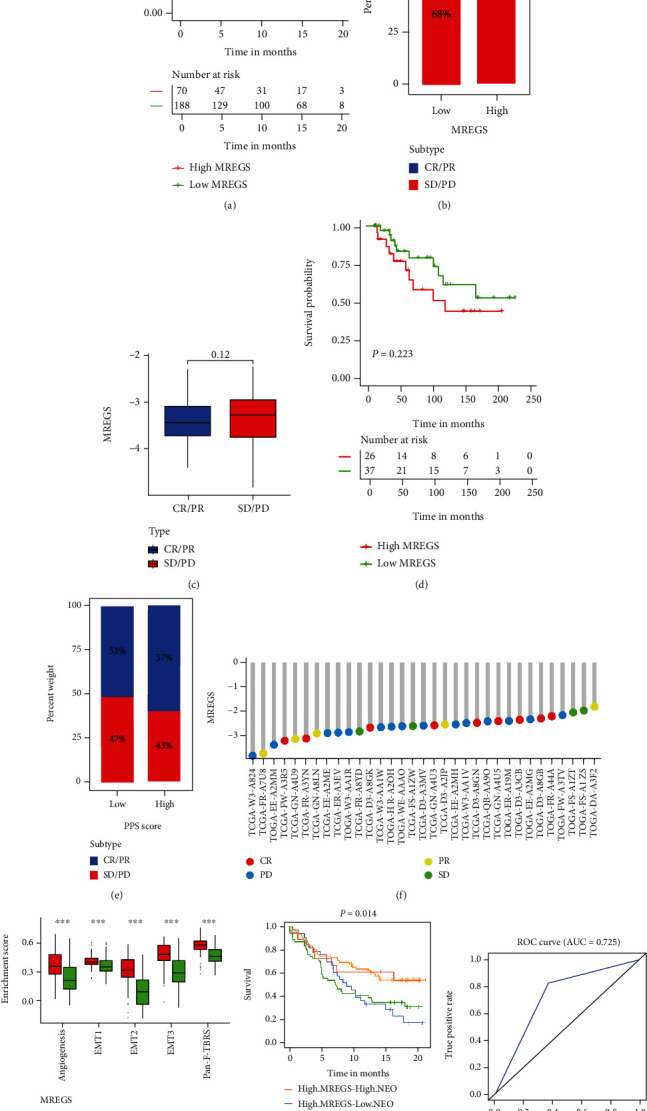
m^6^A-related exosome gene modification patterns in the role of anti-PD-1/L1 immunotherapy. (a) Survival analyses for low (188 cases) and high (70 cases) MREGS patient groups in the anti-PD-L1 immunotherapy cohort using Kaplan-Meier curves (IMvigor210 cohort; *P* = 0.031, log-rank test). (b) The proportion of patients with response to PD-L1 blockade immunotherapy in low or high MREGS groups. SD: stable disease; PD: progressive disease; CR: complete response; PR: partial response; CR/PR: responder; SD/PD: nonresponder. Responder/nonresponder: 32%/68% in the low MREGS groups and 14%/86% in the high MREGS groups. (c) Distribution of MREGS in distinct anti-PD-L1 clinical response groups. (d) Survival analyses for low and high MREGS patient groups in the anti-PD1 immunotherapy cohort using Kaplan-Meier curves (GSE78220 cohort; *P* = 0.223, log-rank test). (e) The proportion of patients with response to PD-1 blockade immunotherapy in low or high MREGS groups. Responder/nonresponder: 53%/47% in the low MREGS groups and 57%/43% in the high MREGS groups. (f) The correlation of MREGS with clinical response to anti-PD-1 immunotherapy. CR, red; PD, blue; PR, yellow; SD, green. (g) Differences in stroma-activated pathways and TGF*β* pathway in fibroblasts between low MREGS and high MREGS groups in the anti-PD-L1 immunotherapy cohort (^∗^*P* < 0.05; ^∗∗^*P* < 0.01; ^∗∗∗^*P* < 0.001). (h) Survival analyses for patients receiving anti-PD-L1 immunotherapy stratified by both MREGS and tumor neoantigen burden using Kaplan-Meier curves. H: high; L: low; NEO: tumor neoantigen burden (*P* < 0.05, log-rank test). (i) The predictive value of the quantification of m^6^A-related exosome gene modification patterns in patients treated with anti-PD-1/L1 immunotherapy (AUC, 0.725).

To test the reliability of MREGS and its prognostic value, MREGS signatures, obtained from the GSE39582 cohort (Figures S7a-b), were also applied to five other colon cancer datasets (GSE17536, GSE29621, GSE33113, GSE37892, and, GSE38832; Figures S7c-g). ROC curve was used to evaluate a model's accuracy, and the higher the AUC value, the more accurate it is. Moreover, MREGS model displayed high accuracy of predictive superiority in patients with 3-year (AUC = 0.785) and 5-year (AUC = 0.754) colon cancer (Figures S7h-i). Immunological therapy exemplified by anti-PD-L1/PD-1 drugs has made immense progress in molecular targeted cancer therapy in recent years. Therefore, we selected two immunotherapy datasets (IMvigor210 and GSE78220) to validate the predictive ability of MREGS to treat patients with PD-L1/PD-1 inhibitors. In the anti-PD-L1 dataset (IMvigor210), through analyzing clinical response and survival rate, we exposed that the low MREGS group surpassed the high MREGS group (Figures 6(a)–6(c)), while in the anti-PD-1 dataset (GSE78220), there was no significant difference between patients with low MREGS and high MREGS (Figures 6(d)–6(f)). The following analysis unraveled that the TME stroma and TGF*β* pathway in fibroblasts were notably activated in the high MREGS group, which mediated tumor migration (Figure 6(g)). Tumor neoantigen burden (TNB) is associated with the efficacy of immunological therapy and is a major element in the judgment of clinical immunotherapies. Furthermore, high TNB would be expected to be characterized by extensive T cell responses and specifically be sensitive to immunotherapy [30]. In addition, we discovered that the low MREGS group with high neoantigens displayed a significant preponderance in survival rate (Figure 6(h)). As aforementioned, MREGS, quantifying m^6^A-related exosome gene signatures, is an underlying and reliable biomarker for evaluating patients' outcomes and clinical manifestation after treating immunological therapy (Figure 6(i)). All in all, calculated from m^6^A-related exosome gene signatures, MREGS is available for forecasting patients' clinical responses to receiving anti-PDL1 drugs and is a referable indicator for the judgment of surgeons.

## 4. Discussion

Increasing evidence illustrated that m^6^A modification played a monumental role in inflammation, innate immunity, and antineoplastic interaction with many m^6^A regulators. However, because most researchers individually paid attention to a kind of immunocyte in a tumor environment or a functioning regulator, the characteristics of TME infiltration were unknown, mediated by the combined function of multiple m^6^A regulators. Exosomes, the vector containing RNA and protein to promote tumor growth, can travel through the whole body. Also, it can deliver “information” from tumors, which can be learned more conveniently than needling biopsy [31, 32]. Thus, through decoding “information” from exosomes in the body fluid, ascertaining the status of distinguishing m^6^A-related exosome gene modification patterns will strengthen the cognitive knowledge of TME immunological reaction and enlighten more targeted immune therapy like personalized treatment.

According to the 59 m^6^A-related exosome genes, TME cell infiltration had distinct characteristics in this study's different m^6^A-related exosome gene modification patterns. Cluster 1, featured by the suppression of immunity, was divided into the immune-desert group. Cluster 2, enriched by natural immunocytes and enhanced activity of stroma, was divided into the immune-excluded group. Cluster 3, which was fulfilled of acquired immune cells in tumors, was divided into the immune-inflamed group. As previous research demonstrated, the scientists would name the tumors in the immune-excluded and immune-desert groups as noninflamed tumors. On the other hand, the tumors in the immune-inflamed group manifested mountainous immune cell infiltration in TME [22, 33, 34]. It is shown that the number of immunocytes was high in the immune-excluded phenotype. However, instead of penetrating the tumor parenchyma, immunocytes tended to retain in the stroma encircled by tumor cells [35–37]. Based on recent reports, increased TGF*β* and activation of EMT-related pathways in the tumor microenvironment would contribute to the immune evasion mechanism that blocks the way to immune infiltration [38, 39]. Some specific molecular inhibitors aim at TGF*β*, which enables the renovation of the tumor microenvironment and liberates T cells from tumors [39, 40]. Furthermore, consistent with the above findings, cluster 2 demonstrated a unique state of stromal activation, with high expression of EMT and TGF pathways and angiogenesis factors, all of which were thought to suppress T cell activation. Therefore, it is ensured that the immune-phenotype classification for three modification patterns is considered credible when connected with each cluster's characteristics of cell infiltration in the tumor microenvironment. According to our findings, there was no doubt that a population of activated innate immunocytes existed in cluster 2 but had a lower survival rate.

Next, mRNA transcriptome differences between distinguishing m^6^A-related exosome gene modification patterns were remarkably relevant to the biological pathways of m^6^A modification, formation of exosomes, and RNA transport. Furthermore, these differentially expressed genes were named m^6^A-related exosome gene signatures. Consistent with the clustering results of the m^6^A modification patterns (clusters 1, 2, and 3), three gene clusters (A, B, and C) clustered by m^6^A-related exosome gene signatures were also significantly correlated with stromal activation and immune response. Hence, it will increase our knowledge of TME cell-infiltrating characteristics by comprehensively assessing m^6^A-related exosome gene modification patterns. Due to the difference in m^6^A modification in different tumors, every tumor should be quantified its unique m^6^A modification patterns. Therefore, we set a scoring role to quantify the level of m^6^A methylation in exosomes of sufferers with colon cancer—m^6^A-related exosome gene score (MREGS). As mentioned, the immune-excluded-related modification pattern displayed a higher MREGS, whereas lower MREGS was exhibited in the immune-inflamed-related pattern. It indicated that MREGS is one of the reliable indexes to evaluate the m^6^A-related exosome gene modification pattern of individual tumor and further identify tumor immune phenotype.

Our data also illustrated that low MREGS strongly correlated with high tumor mutation burden (hTMB), developing a more robust and sensitive biomarker to immune checkpoint inhibitors and a positive index to receive immunotherapeutic treatments like immune checkpoint inhibitor (ICPI) [41]. In addition, the low MREGS group was related to the dMMR (different mismatch repair) subtype. Previous reports said, metastatic colorectal cancer patients with dMMR could benefit tremendously from immune checkpoint inhibitors such as PD-L1 monoclonal antibody pembrolizumab [42]. Moreover, we found that the low MREGS group with high neoantigens had higher survival rates than the high MREGS group with high neoantigens. Furthermore, Anagnostou et al. discovered that as more neoantigens were identified, the immune system increased T cells fighting tumors, increasing the efficacy of ICPI [43]. Therefore, MREGS with integrated biomarkers like mutation load, neoantigen load, MMR status, stromal activation, and TME immune phenotypes could be more beneficial for designing strategies for immunotherapy. Also, in the cohort with anti-PD-L1 immune therapy, the predictive value of MREGS was confirmed, and a remarkable difference existed in MREGS between responders and nonresponders.

Interestingly, analyzing the correlation between adjuvant chemotherapy (ADJC) and MREGS, the high MREGS group was more suitable for ADJC than the low MREGS group because ADJC negatively impacted the low MREGS group reflecting in survival rate. Combined with two facts that high MREGS was relevant to the augmented expression of TGF*β* and EMT and a higher possibility of tumor recurrence in patients with increased TGF*β*- and EMT-related pathways [44], it is not hard to conclude that ADJC can restrain the metastasis of residual cancer cells. However, ADJC also had certain toxicity on patients as the lower survival rate in low MREGS with the ADJC group versus with the non-ADJC group. Consequently, MREGS is probably expected to be a biomarker to sift optimal sufferers that need to receive ADJC-like microsatellite status and BRAF and KRAS mutations [45].

In sum, detecting the level of m^6^A methylation of exosomes in patients' serum, MREGS could be utilized for assessing the m^6^A-related exosome gene modification patterns of each patient and their matching characteristics of TME cell infiltration. Next, ascertain immunological classifications of tumors and assist clinicians in tailoring optimal treatment for patients. We also expounded that MREGS could evaluate patients' clinicopathological features such as genetic variation, the status of tumor cell infiltration, MMR status, clinical stages, and TMB. Our analysis graphics could find the correlation between MREGS and clinicopathological characteristics. Furthermore, predicting the clinical reaction to anti-PDL1 immune therapy and the therapeutic effect of ADJC will be one of MREGS's greatest advantages. Although this approach was convenient for detection, it also had several unresolved challenges, such as exosome extraction and purity, the sensitivity of detected m^6^A methylation, and how to deal with cancer metastasis, which meant lesions were not only found in one tissue of one patient. Current solutions, in our opinion, are constantly reforming exosome extraction technologies and platforms and deeply mining clinical data to explore tumor-specific exosome markers.

## Data Availability

Publicly available datasets or databases were analyzed in the present study. This data can be found here: TCGA database (https://portal.gdc.cancer.gov) and GEO database (https://www.ncbi.nlm.nih.gov/geo/), under the accession numbers GSE17536, GSE29621, GSE33113, GSE37892, GSE38832, and GSE39582.

## Supplementary Materials

Supplementary Materials 1 Supplementary methods. Supplementary figure 1: overview of study design and prognostic analysis of 59 m^6^A-related exosome genes. Supplementary figure 2: unsupervised clustering of 59 m^6^A-related exosome genes in the 6 independent colon cancer cohorts. Supplementary figure 3: the correlation between each TME infiltration cell type and each m^6^A-related exosome gene using Spearman analyses. Supplementary figure 4: unsupervised clustering of 59 m^6^A-related exosome genes in the GSE39582 colon cancer cohort. Supplementary figure 5: unsupervised clustering of 3787 m^6^A phenotype-related exosome genes in GSE39582 cohort. Supplementary figure 6: the prognostic value of MREGS and correlation between the clinicopathological features and MREGS. Supplementary figure 7: prognostic value of MREGS in colon cancer cohorts.
