# Supplementary material for: m6A Regulator-Based Exosomal Gene Methylation Modification Patterns Identify Distinct Microenvironment Characterization and Predict Immunotherapeutic Responses in Colon Cancer
Source: Oxid Med Cell Longev. 2022 Aug 22;2022:9451480. doi: 10.1155/2022/9451480 (PMC9423980; doi:10.1155/2022/9451480)
Supplement: Supplementary Materials — 1 Supplementary methods. Supplementary figure 1: overview of study design and prognostic analysis of 59 m6A-related exosome genes. Supplementary figure 2: unsupervised clustering of 59 m6A-related exosome genes in the 6 independent colon cancer cohorts. Supplementary figure 3: the correlation between each TME infiltration cell type and each m6A-related exosome gene using Spearman analyses. Supplementary figure 4: unsupervised clustering of 59 m6A-related exosome genes in the GSE39582 colon cancer cohort. Supplementary figure 5: unsupervised clustering of 3787 m6A phenotype-related exosome genes in GSE39582 cohort. Supplementary figure 6: the prognostic value of MREGS and correlation between the clinicopathological features and MREGS. Supplementary figure 7: prognostic value of MREGS in colon cancer cohorts. [file 9451480.f1.docx]

**Supplementary methods**

**Gene set variation analysis (GSVA) and functional annotation**

To investigate the difference in biological process between m^6^A-related exosome gene modification patterns, we performed GSVA enrichment analysis using "GSVA" R packages. GSVA, in a non-parametric and unsupervised method, is commonly employed for estimating the variation in the pathway and biological process activity in the samples of an expression dataset. The gene sets of "c2.cp.kegg.v6.2.- symbols" were downloaded from the MSigDB database for running GSVA analysis. Adjusted P with a value less than 0.05 was considered statistically significant. The clusterProfiler R package was used to perform functional annotation for m^6^A-related exosome genes, with the cutoff value of FDR < 0.05. Estimating of TME cell infiltration, we used the ssGSEA (single-sample gene-set enrichment analysis) algorithm to quantify the relative abundance of each cell infiltration in CC TME. The enrichment scores calculated by ssGSEA analysis were utilized to represent the relative abundance of each TME infiltrating cell in each sample

**Identification of differentially expressed genes (DEGs) between m^6^A-related exosome gene distinct phenotypes**

To identify m^6^A-related exosome genes, we classified patients into three distinct m^6^A -related exosome modification patterns based on the expression of 59 m^6^A-related exosome genes. The empirical Bayesian approach of the limma R package was applied to determine DEGs between different modification patterns. The significance criteria for determining DEGs were adjusted (P value < 0.001).

**Generation of m^6^A-related exosome gene signature**

To quantify an individual tumor's m^6^A-related exosome gene modification patterns, we constructed scoring rules to evaluate the m^6^A modification pattern of individual patients with colon cancer—the m^6^A-related exosome gene signature. The procedures for establishing m^6^A-related exosome gene signature were as follows: The DEGs identified from different m^6^A clusters were first normalized among all GSE39582 samples and the overlap genes were extracted. Next, the patients were classified into several groups for deeper analysis by adopting an unsupervised clustering method for analyzing overlapped DEGs. The consensus clustering algorithm was utilized fto define the number of gene clusters and their stability. Then, we performed the prognostic analysis for each gene in the signature using a univariate Cox regression model. The genes with a significant prognosis were extracted for further analysis. We then conducted principal component analysis (PCA) to construct a m^6^A-related exosome gene signature. Both principal components 1 and 2 were selected to act as signature scores. This method had the advantage of focusing the score on the set with the largest block of well correlated (or anticorrelated) genes while down-weighting contributions from genes that do not track with other set members. We then define the MREGS using a method like GGI. Finally, we performed a correlation analysis to reveal further association between m^6^A-related exosome gene signature and some related biological pathways.

**Collection of immune-checkpoint blockade genomic and clinical information**

We systematically searched the immune checkpoint blockade gene expression profiles, which could be publicly obtained and reported with complete clinical information. Our study finally included two immunotherapeutic cohorts: advanced urothelial cancer with the intervention of atezolizumab, an anti-PD-L1 antibody (IMvigor210 cohort). For IMvigor210 cohort, based on the Creative Commons 3.0 License, the complete expression data and detailed clinical annotations could be obtained from http://research-pub.Gene.com/imvigor210corebiologies. Finally, the raw data were normalized using the DEseq2 R package and then the count value was transformed into TPM value.

**Supplementary figures and legends**


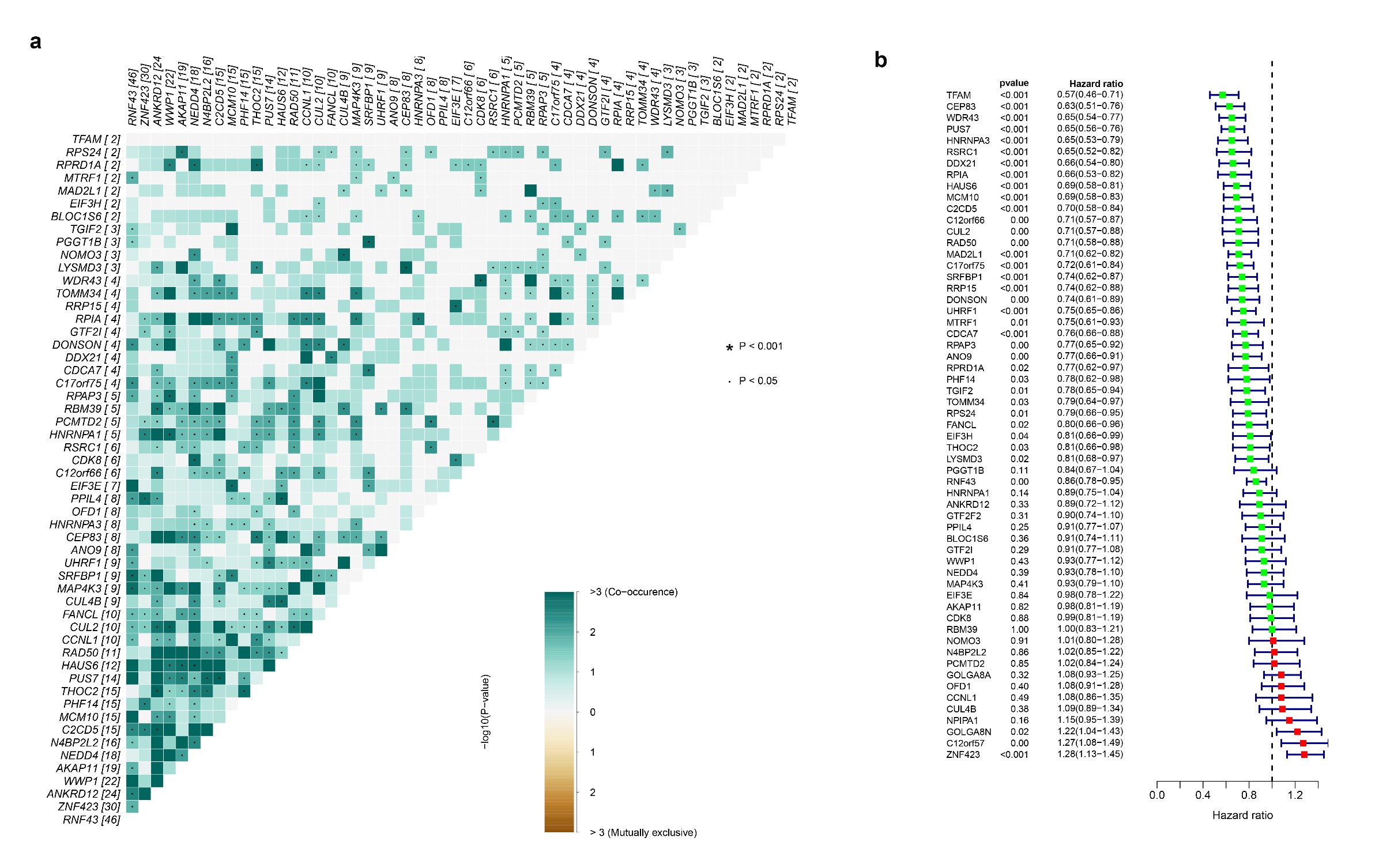


**Figure S1 Overview of study design and prognostic analysis of 59 m^6^A-related exosome genes**

(a) The mutation co-occurrence and exclusion analyses for 59 m^6^A-related exosome genes. Co-occurrence, green; Exclusion, brown. (b) The prognostic analyses for 59 m^6^A-related exosome genes in the colon cancer cohorts using a univariate Cox regression model. Hazard ratio >1 represented risk factor for survival and hazard ratio＜1 represented protective factor for survival.


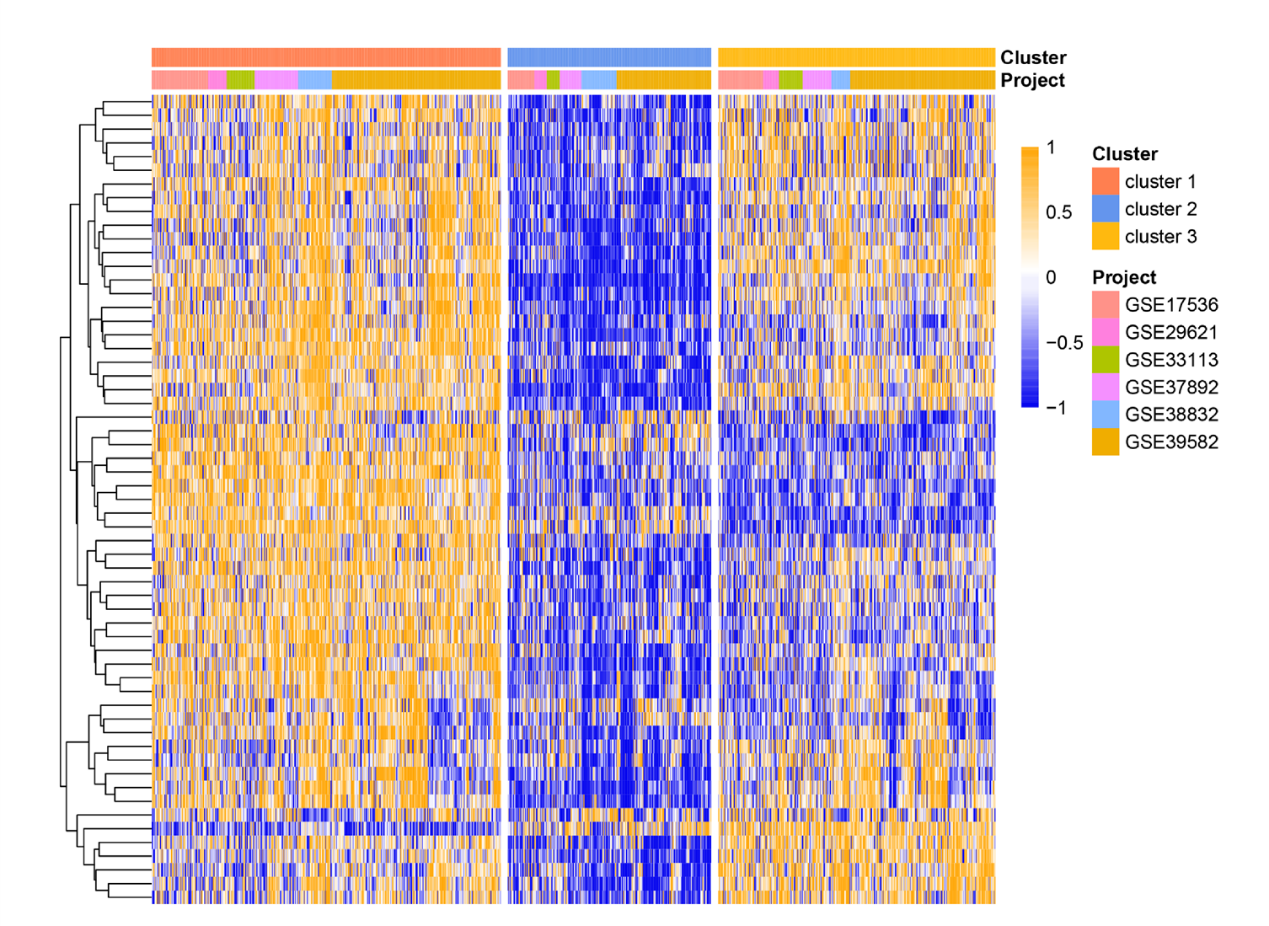


**Figure S2 Unsupervised clustering of 59 m^6^A-related exosome genes in the 6 independent colon cancer cohorts.**

The clusters and cohort names were used as patient annotations. Each column represented patients and each row represented m^6^A-related exosome genes.


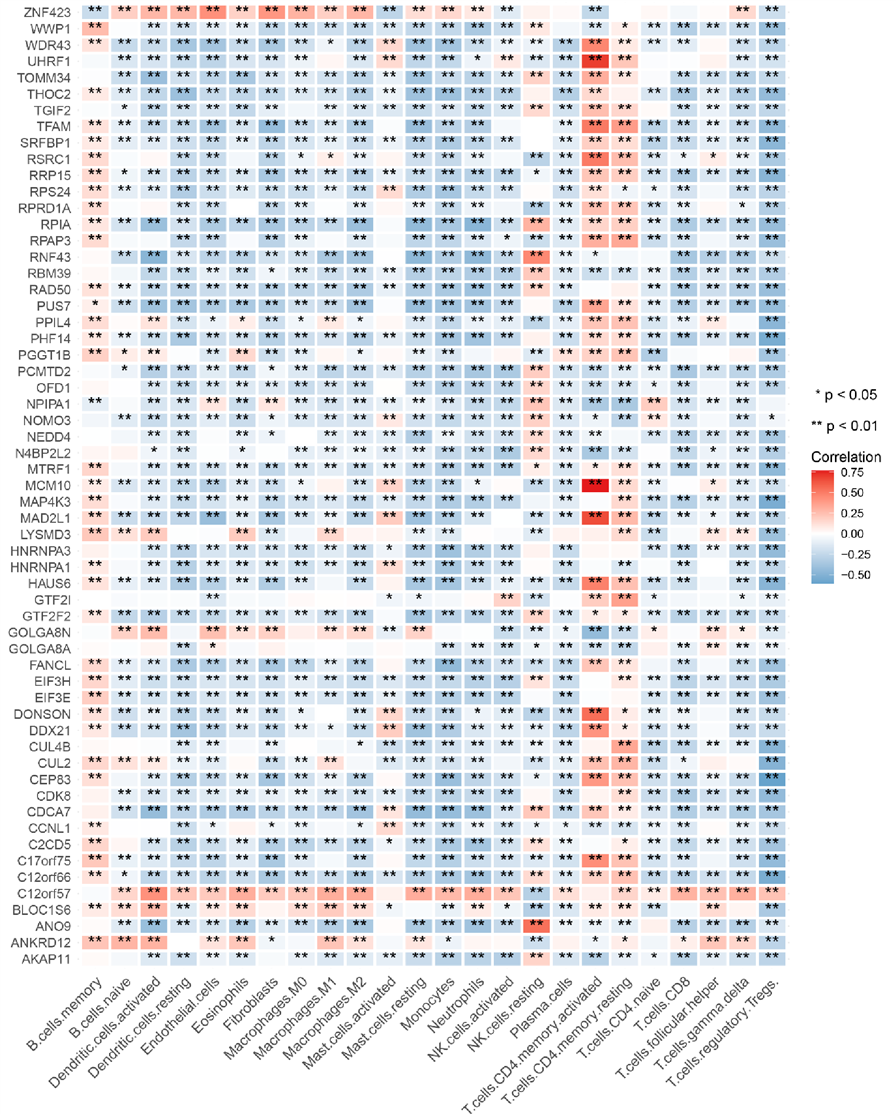


**Figure S3 The correlation between each TME infiltration cell type and each m^6^A-related exosome gene using spearman analyses.**

Negative correlation was marked with blue and positive correlation with red. (*P < 0.05; **P < 0.01)


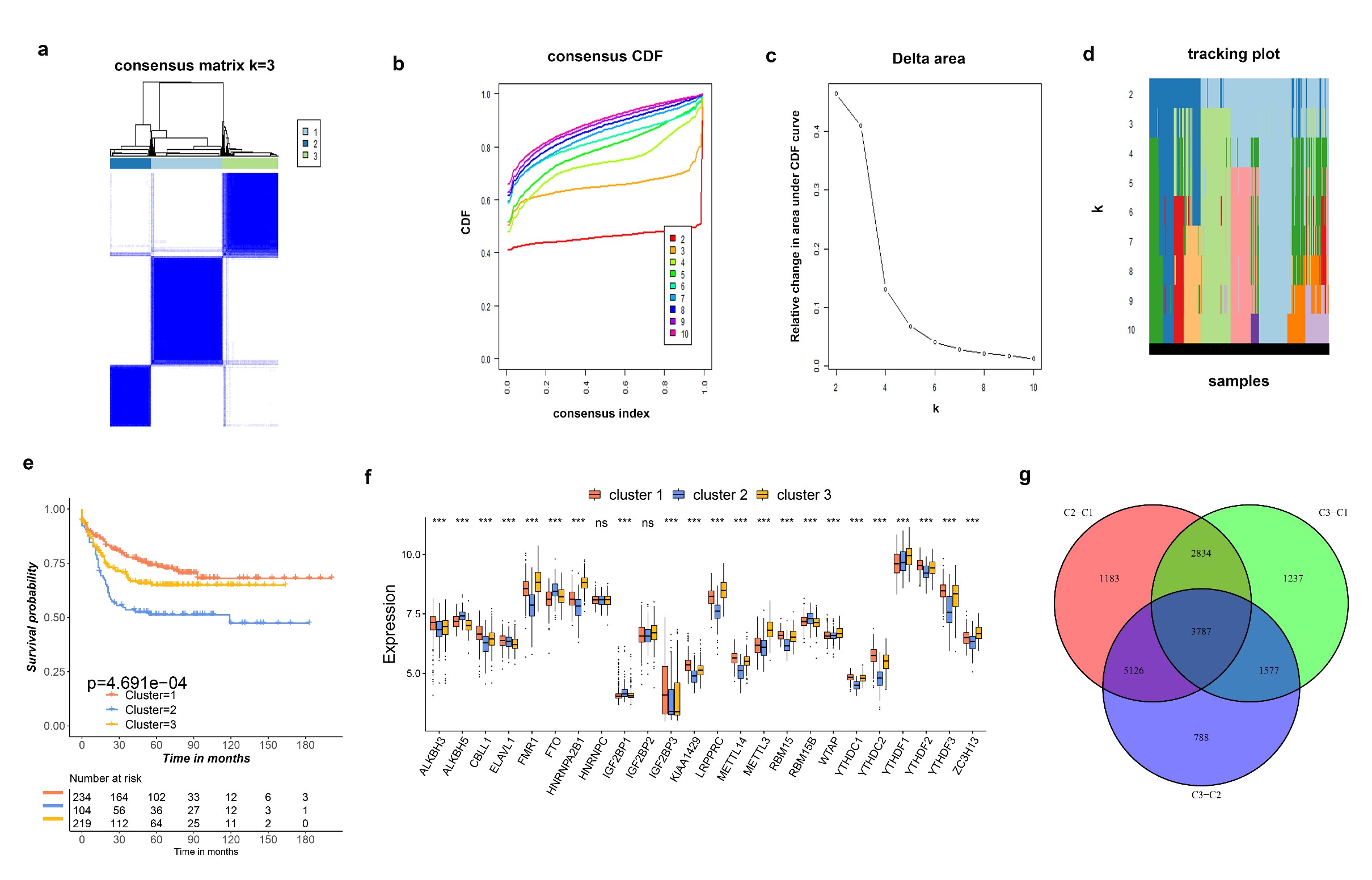


**Figure S4. Unsupervised clustering of 59 m^6^A-related exosome genes in the GSE39582 colon cancer cohort.**

(a) Consensus matrices of the GSE39582 cohort for k = 3. (b)consensus CDF (c)delta area (d)tracking plot (e)Survival analyses for the three m^6^A-related exosome gene modification patterns in GSE39582 cohort using Kaplan-Meier curves including 234 cases in cluster 1, 104 cases in cluster 2, and 219 cases in cluster 3. The cluster 1 showed significantly better overall survival than the other two clusters. (p＜0.05, Log-rank test) (f) The expression of 24 m^6^A regulators genes in the three clusters. The upper and lower ends of the boxes represented interquartile range of values. The lines in the boxes represented median value, and black dots showed outliers. The asterisks represented the statistical p value. (*P < 0.05; **P < 0.01; ***P < 0.001) (g) 3787 m^6^A phenotype-related exosome genes shown in Venn diagram.


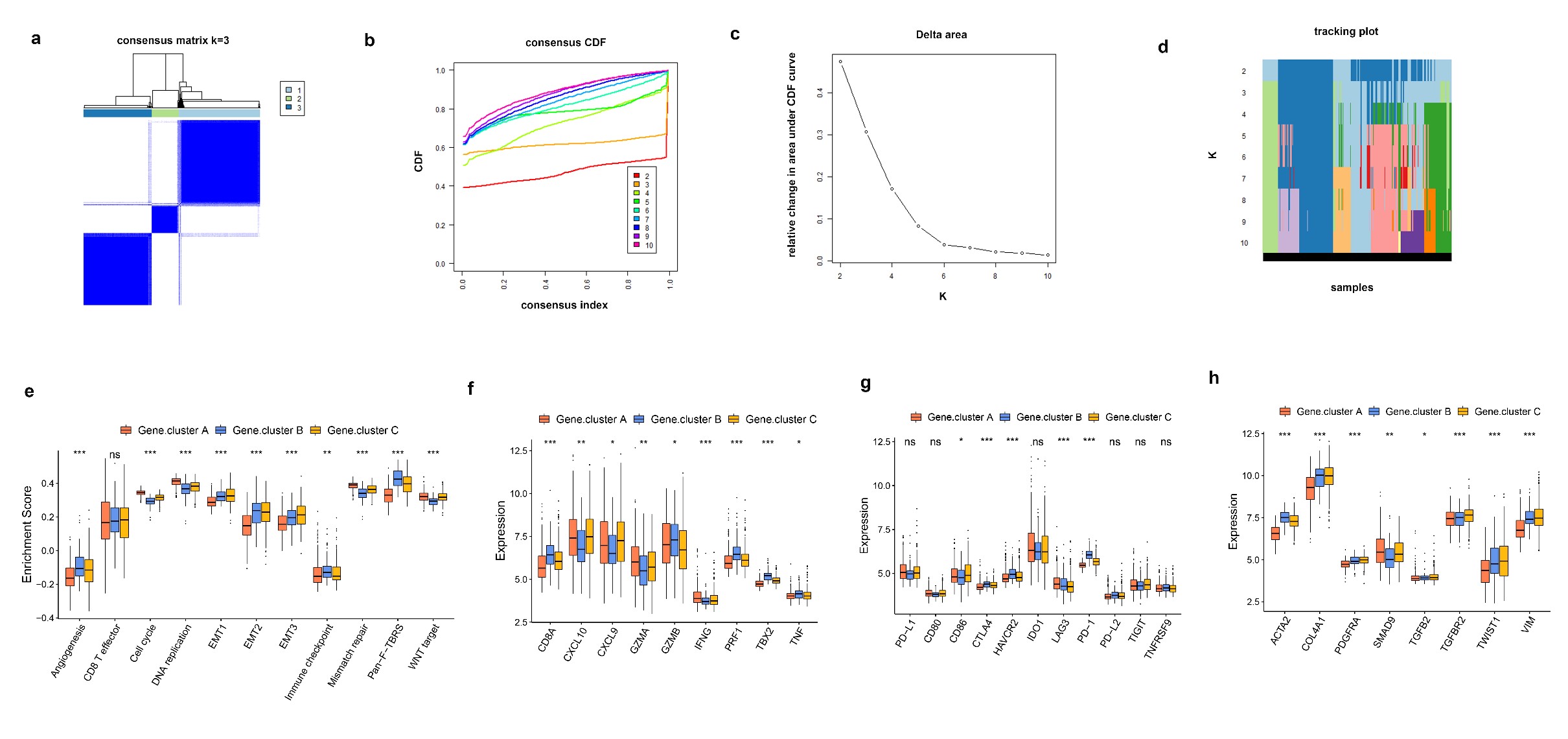


**Figure S5 Unsupervised clustering of 3787 m^6^A phenotype-related exosome genes in GSE39582 cohort.**

(a) Unsupervised clustering of 3787 m^6^A phenotype-related exosome genes in GSE39582 cohort and consensus matrices for k = 3. (b)consensus CDF (c)Delta area (d)tracking plot (e) Difference in the expression of known signatures including stromal-activation related signatures, tumor-promotion related signatures and immune-activation related signatures among three exosome gene clusters. The upper and lower ends of the boxes represented interquartile range of values. The lines in the boxes represented median value, and black dots showed outliers. The asterisks represented the statistical p value. (*P < 0.05; **P < 0.01; ***P < 0.001) (f-h) Difference in the immune-activation related gene expression among three gene clusters.


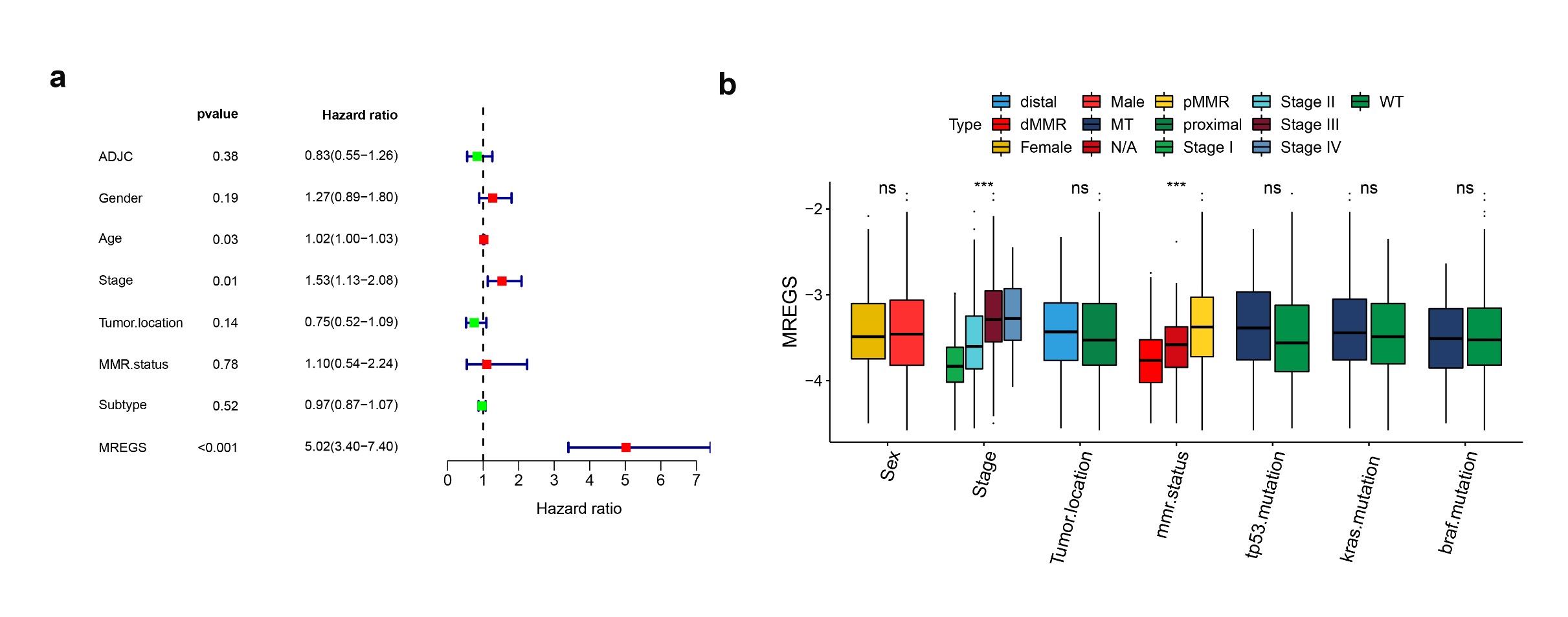


**Figure S6 The prognostic value of MREGS and correlation between the clinicopathological features and MREGS**

(a) Multivariate Cox regression analysis for MREGS in GSE39582 cohort shown by the forest plot. (b) Difference in MREGS among distinct clinical subgroups in GSE39582 cohort. ADJC, adjuvant chemotherapy. (*P < 0.05; **P < 0.01; ***P < 0.001).


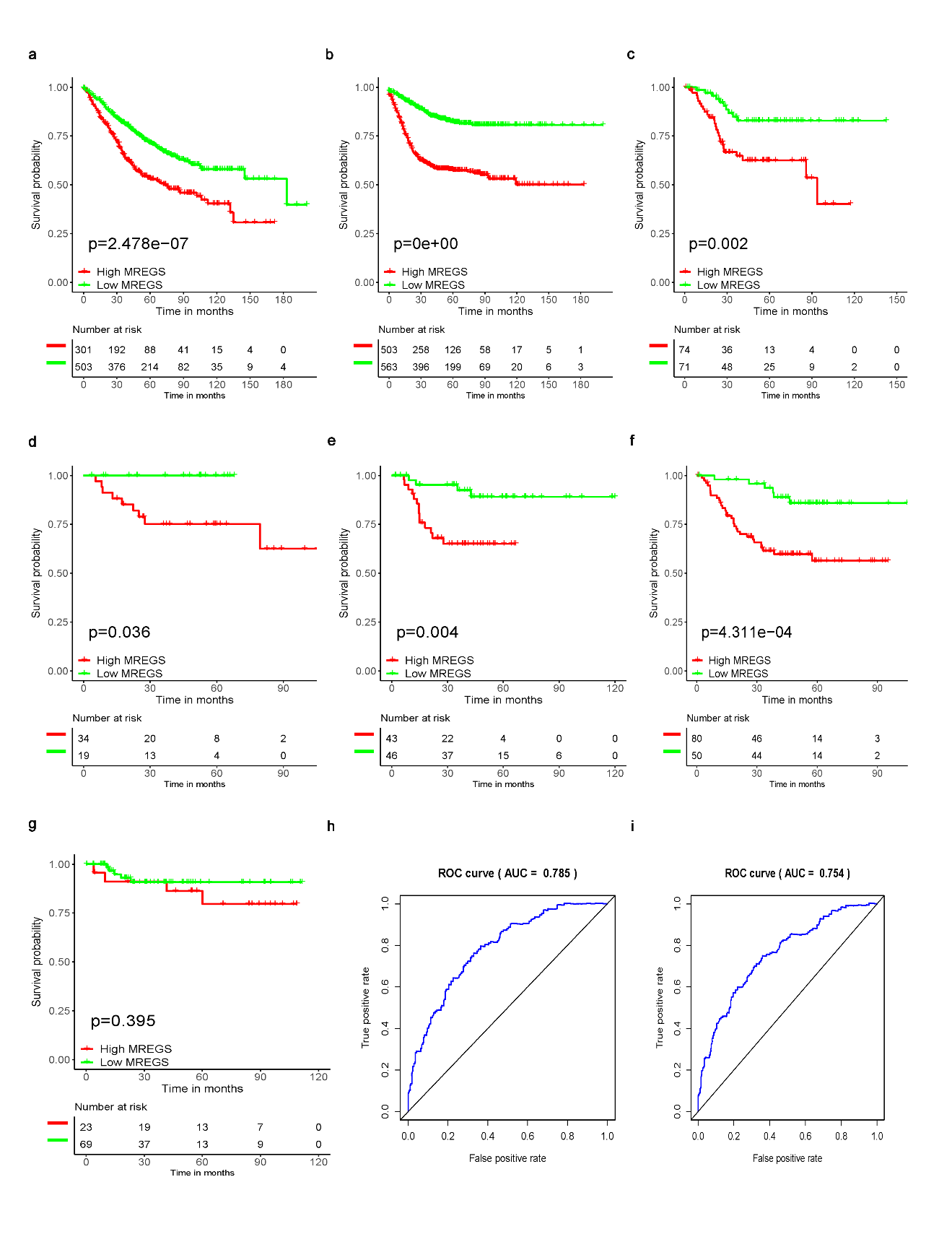


**Figure S7 Prognostic value of MREGS in colon cancer cohorts**

(a) GSE39582(OS, overall survival): P < 0.0001, Log-rank test. (b) GSE39582(RFS, recurrence free survival): P < 0.0001, Log-rank test. (c) GSE17536(OS, overall survival): P =0.002, Log-rank test. (d) GSE29621(OS, overall survival): P =0.036, Log-rank test. (e) GSE33113(OS, overall survival): P =0.004, Log-rank test. (f) GSE37892(OS, overall survival): P < 0.0001, Log-rank test. (g) GSE38832(OS, overall survival): P =0.395, Log-rank test. (h)The predictive value of MREGS in patients with 3-year colon cancer. AUC, 0.785. (i) The predictive value of MREGS in patients with 5-year colon cancer. AUC, 0.754.
